# Role for diet in normal gut barrier function: developing guidance within the framework of food-labeling regulations

**DOI:** 10.1152/ajpgi.00063.2019

**Published:** 2019-05-24

**Authors:** Michael Camilleri, Barbara J. Lyle, Karen L. Madsen, Justin Sonnenburg, Kristin Verbeke, Gary D. Wu

**Affiliations:** ^1^Clinical Enteric Neuroscience Translational and Epidemiological Research, Division of Gastroenterology and Hepatology, Mayo Clinic, Rochester, Minnesota; ^2^International Life Sciences Institute North America, Washington, DC; ^3^School of Professional Studies, Northwestern University, Evanston, Illinois; ^4^Department of Medicine, University of Alberta, Edmonton, Alberta, Canada; ^5^Department of Microbiology and Immunology, Stanford University School of Medicine, Stanford, California; ^6^Translational Research in Gastrointestinal Disorders, Katholieke Universiteit Leuven, Leuven, Belgium; ^7^Division of Gastroenterology, Perelman School of Medicine, University of Pennsylvania, Philadelphia, Pennsylvania

**Keywords:** diet, function, gut barrier structure, permeability

## Abstract

A reduction in intestinal barrier function is currently believed to play an important role in pathogenesis of many diseases, as it facilitates passage of injurious factors such as lipopolysaccharide, peptidoglycan, whole bacteria, and other toxins to traverse the barrier to damage the intestine or enter the portal circulation. Currently available evidence in animal models and in vitro systems has shown that certain dietary interventions can be used to reinforce the intestinal barrier to prevent the development of disease. The relevance of these studies to human health is unknown. Herein, we define the components of the intestinal barrier, review available modalities to assess its structure and function in humans, and review the available evidence in model systems or perturbations in humans that diet can be used to fortify intestinal barrier function. Acknowledging the technical challenges and the present gaps in knowledge, we provide a conceptual framework by which evidence could be developed to support the notion that diet can reinforce human intestinal barrier function to restore normal function and potentially reduce the risk for disease. Such evidence would provide information on the development of healthier diets and serve to provide a framework by which federal agencies such as the US Food and Drug Administration can evaluate evidence linking diet with normal human structure/function claims focused on reducing risk of disease in the general public.

## INTRODUCTION: OBJECTIVES

This perspective article summarizes the present scientific evidence focused on *1*) the gut barrier as an important component of normal gastrointestinal (GI) structure and function in human health as well as identification of specific physiologic benefits causally associated with normal structure and function, *2*) currently available modalities to describe the intestinal barrier and quantify its function in humans, and *3*) providing possible associations between diet and normal gut barrier function among healthy or at-risk people. It is intended to inform translational research into dietary guidance (such as food-labeling claims) as well as address human nutrition research design decisions so that evidence is useful for making dietary guidance decisions. Despite a tremendous amount of interest in the notion that the modalities to reduce the “leaky gut” may reduce the risk for disease, there are significant limitations in our present understanding of human intestinal barrier function, as well as technical challenges that must be addressed to advance the field forward in a rigorous and reproducible scientifically based manner. Gaps and means to address them are featured throughout, with the intention to inform future translational research to serve as the basis of dietary guidance broadly.

Advances in this field of research will be fundamentally important in many practical issues that will be very impactful to human health. For example, they will have an impact on the manner in which we apply the framework established by the US Food and Drug Administration for evaluating evidence linking diet with normal human structure/function and with reducing risk of disease or medical outcomes as they are related to the general public (208, 209). This is particularly relevant, given the new definition of dietary fiber for food labeling in the United States that requires a demonstrated physiological effect that is beneficial to human health [either structure/function (e.g., laxation) or specific health-related biomarkers (e.g., inflammation)]. Table 1 describes how nutrition research evidence applies to maintaining normal gut barrier structure and function, as well as reducing the risk of gut barrier-related health conditions such as bowel diseases. Such structure/function claims are regulated by the US Food and Drug Administration: claims for conventional foods focus on effects derived from nutritive value, whereas claims for dietary supplements may focus on nonnutritive as well as nutritive effects. This review is necessitated by the recent interest in intestinal barrier function and leaky gut in diverse GI and non-GI diseases and anticipated claims of the structure/function effects of different nutrients or other remedies. This article is a distillation of presentations at a meeting organized by International Life Sciences Institute North America in December 2018.

**Table 1.** Framework to evaluate scientific evidence linking diet with gut barrier structure, function, and risk reduction for adverse bowel conditions

Evidence That Would Apply If Available	Structure and Function Claims	Health Claims
Examples of messages associated with structure/function claims	*Dietary component X* helps maintain normal gut barrier structure; *dietary component X* helps maintain a normally functioning gut barrier; limiting *dietary component X* helps maintain a normally functioning gut barrier; *dietary component X* helps maintain normal nutrient absorption while protecting against harmful exposures in the gut	*Dietary component X* helps reduce risk of [insert disease or health-related condition Y*]; for example, reduced risk of IBD among persons with a family history or [insert other conditional factors defining the relevant population]
Primary evidence	Human studies demonstrating that *dietary component X* is causally associated with maintaining or restoring normal gut barrier structure (e.g., mucus layer thickness) or function of human intestinal barrier (e.g., “normal” permeability or epithelial cell immune function); human studies demonstrating a physiological benefit to normal gut barrier permeability and gut immunological function (e.g., reduced susceptibility to food-borne/intestinal pathogens or preventing elevated endotoxins or systemic inflammation)	Strength of evidence from human studies demonstrating a clinically and statistically significant relationship between the dietary component and accepted indicators of risk for or progression to [insert specific intestinal or extraintestinal health conditions such as IBD or metabolic syndrome]
Background information	Animal studies that link *1*) structure with normal permeability or other to-be-determined indicators of normal function (e.g., preventing chronic systemic inflammation and maintaining immune components in gut membranes) or *2*) normal structure or function with a specific health benefit (such as protection from food-borne/intestinal pathogen exposure); human mechanism-of-action studies supporting a causal link between dietary component and structure/function of gut barrier (e.g., increased fermentable fiber, which protects mucin layer thickness)	Animal studies showing that *dietary component X* reduces risk or surrogate markers of *disease or health-related condition Y*; human mechanism-of-action studies associating dietary component with disease risk or surrogate marker of disease risk; human mechanism-of-action studies to support a causal relationship
Surrogate measure in human studies		Identify clinically accepted indicators of risk by specific condition identified above (e.g., include a gut example…similar to how elevated LDL cholesterol is recognized by the FDA as a risk factor for cardiovascular disease)

* Examples listed here require US Food and Drug Administration (FDA) review to evaluate whether they meet regulatory requirements of a disease or health-related condition: helps reduce risk of inflammatory bowel disease (IBD), helps reduce risk of celiac disease, helps reduce susceptibility to or development of food allergies, helps reduce risk of metabolic dysfunction, helps prevent low weight for age among children at risk for gastrointestinal disease-induced malnourishment, and helps reduce risk of environmental enteric dysfunction among young children exposed to environmental risks.

## STRUCTURAL AND FUNCTIONAL COMPONENTS OF THE INTESTINAL BARRIER

Barrier function at a mucosal interface is an essential property of host biology that provides protection from the environment. The intestinal epithelium forms a dynamic and semipermeable barrier that allows the absorption of nutrients, electrolytes, and water, as well as antigens that play a role in immune regulation, but it also protects the host from the microbiota, as well as potentially toxic molecules in the gut lumen. Since intestinal mucosal barrier dysfunction is associated with numerous diseases, it is reasonable to postulate that its reinforcement in health may reduce the risk of disease. Disease entities where this might be the case include those that are specific to the intestinal tract such as ulceration, enteric infections, functional bowel disorders, cancer, and intestinal immune-mediated disorders such as inflammatory bowel or celiac disease. Barrier function also appears to differ with age (discussed below in potential of dietary and nondietary factors to impact components of the intestinal mucosal barrier, *Diet-Independent Factors Affecting Intestinal Permeability*, *Age*).

**Fig. 1.**
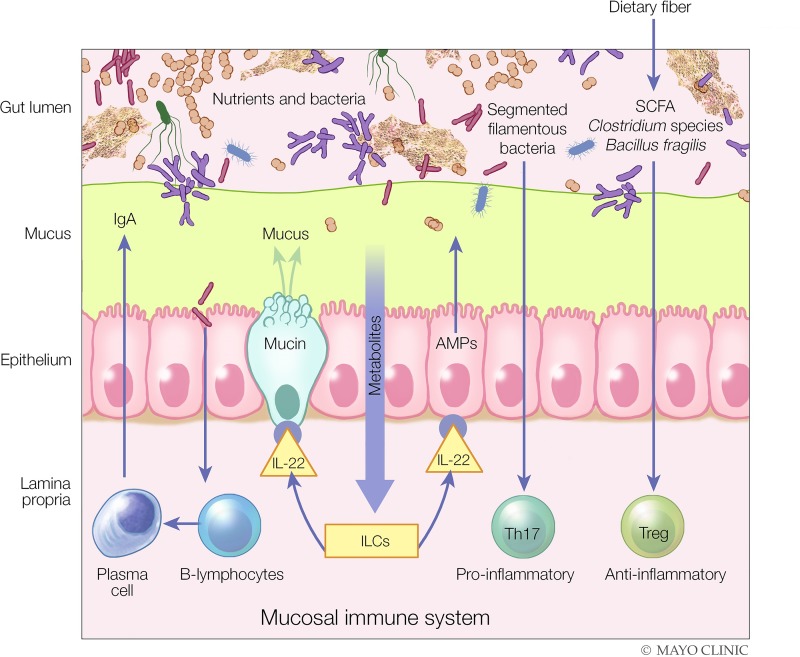
The three components of the intestinal mucosal barrier and the impact of diet and specific immune mechanisms involved in maintaining the integrity of the barrier. Diet can reinforce both the structure and function of the intestinal barrier, for example, through the production of short-chain fatty acids (SCFAs) by the gut microbiota, which are used by the colonic epithelium as a source of energy and can, independently, induce immune tolerance via T regulatory (Treg) cells. As another example, metabolites in diet can activate innate lymphoid cells (ILCs) to produce IL-22, which, in turn, can enhance the production of mucin and antimicrobial peptides (AMPs) by the intestinal epithelium to fortify gut barrier function. Plasma cells, a component of the mucosal immune system, can also produce IgA, which is secreted into the intestinal mucus layer. In this manner, the intestinal epithelium, the most important component of the intestinal barrier, has both structural and functional components to protect the host from the luminal contents of the intestinal tract. Th17, T helper type 17. [Modified from Kamada and Núñez (108) with permission.]

The mucosal barrier can be divided roughly into three interacting components: mucus, the intestinal epithelium, and the mucosal immune system [Fig. 1 (108)]. Intestinal mucus is a gel layer composed of complex glycoproteins important as the first-line barrier of the mucosal surface but also serving as a lubricant and aiding as a transport vehicle between luminal contents and the intestinal epithelium. In the colon, there are two mucus layers, a loose outer layer that is inhabited by the gut microbiota where it is utilized as a source of bacterial nutrition and a dense inner layer that is largely devoid of bacteria [reviewed by Johansson et al. (101)].

Mucus has potent antimicrobial activity because it contains antimicrobial peptides (AMPs) such as α- and β-defensins that are secreted by intestinal epithelial cell lineages such as Paneth cells (153) and secretory IgA produced by plasma cells located in the lamina propria. Secretory IgA is the most abundant immunoglobulin at mucosal surfaces (155), where it plays a significant role in maintaining barrier function within the intestinal mucus by binding to bacteria in the gut lumen with some level of specificity and preventing microbial invasion by coating bacteria, inhibiting adherence to epithelial cells, and neutralizing bacterial toxins.

The most important physical barrier at the intestinal mucosal surface lies underneath the mucus layer, the intestinal epithelium. These cells form a continuous and polarized epithelial barrier with several cellular lineages: absorptive cells, goblet cells, enteroendocrine cells, tuft cells, and Paneth cells. Each cell type has specific functions. For example, both goblet cells and Paneth cells help to fortify the mucosal barrier through the production of mucus and AMPs, respectively. Substances can pass through this barrier via two general pathways, transcellular or paracellular. Transcellular permeability is associated with solute transport predominantly regulated by selective transporters, such as the sodium-coupled transporters for glucose and fructose (85, 140), as well as neutral, dibasic, and dicarboxylic amino acids (1), whereas paracellular permeability is associated with transport in the space between epithelial cells and is regulated by intercellular complexes localized toward the apical surface of the intestinal epithelium (5, 17). These intercellular complexes are also under neurohumoral control, such as from vasoactive intestinal peptide and cholinergic neurons from the submucosal plexus (79, 82, 166). However, it is unclear whether specific transporters exist for some of the disaccharide molecules used in the measurement of intestinal permeability, such as lactulose and sucralose.

Located primarily below the intestinal epithelium, with some components situated in between, is the immunological barrier of the intestinal mucosa, known as the mucosal immune system, the largest immune organ in the human body. A complex and interconnected array of various immune cell populations maintains intestinal mucosal immune homeostasis by balancing immune activation to protect against microbial invasion while simultaneously demonstrating restraint via immune tolerance to prevent unrestrained inflammation, the hallmark of immune-mediated intestinal diseases such as inflammatory bowel diseases (147). General populations of the intestinal immune effector system include T effector and regulatory cells, B cells, dendritic cells, and components of the innate immune system such as macrophages, mast cells, and neutrophils [reviewed by Veldhoen and Brucklacher-Waldert (215)]. An important and more recently described cell population is the innate lymphoid cells (ILCs), which belong to the lymphoid lineage but lack antigen-specific T and B cell receptors (147, 215).

Interaction between all three components of the intestinal mucosal barrier is complex but is well orchestrated to maintain a homeostatic state between the host and its environment at the mucosal interface. For example, ILC3s are particularly important in the maintenance of the intestinal mucosal barrier through the secretion of IL-22 cytokine, which promotes both enhanced mucus production via goblet cells as well as AMPs. Both of these are important components of the intestinal mucus barrier. Through this multicomponent intestinal barrier system, a normally functioning gut barrier selectively enables absorption of water and essential nutrients, while protecting against adverse health effects from ingested or endogenous toxins.

## MEASUREMENTS TO CHARACTERIZE NORMAL STRUCTURE AND FUNCTION OF THE INTESTINAL BARRIER IN HUMAN NUTRITION RESEARCH

Gut permeability is a useful measure of how well the integrated system of the GI barrier (summarized as surface mucus, epithelial barrier, and immune mechanisms) is functioning. The goal in measuring intestinal permeability is to document dysfunction or leakiness of the barrier, with published research focusing on its association with risk of intestinal diseases such as celiac disease and inflammatory bowel disease, which are associated with clear morphological changes in the three components of the intestinal barrier. Increased permeability alone may not be the causal or strong contributing factor in the disease (150). It is presently unclear whether normal permeability can become “tighter” and whether doing so would enhance a healthy state.

The focus of nutrition should be on factors that maintain or restore normal gut barrier structure and function. Thus, this section addresses the optimal methods of measurement (focused on humans) and barrier function in the absence of overt mucosal inflammation or ulceration. Therefore, it is relevant to examine barrier function in health but even more in stressed conditions such as food allergy or intolerance, or irritable bowel syndrome (IBS), in which there is evidence of immune activation without significant inflammation or ulceration. This would be analogous to studying diet effects in overweight people [body mass index (BMI) 25–30 kg/m^2^] rather than the clinically diagnosed disease, obesity (BMI >30 kg/m^2^).

### Intestinal Permeability Measurement

Table 2 compares methods for measuring GI structure and function in the assessment of barrier function (23). In vitro methods, such as the Ussing chamber technique, quantify mucosal to serosal fluxes of probe molecules across mucosal biopsies obtained from different regions of the gut but require multiple mucosal biopsies at different levels of the gut and are, therefore, too invasive for most human research (83). Thus, human intestinal permeability is studied predominantly by measuring the urine excretion of orally consumed probe molecules (17) and by direct measurements during endoscopic procedures.

**Table 2.** Techniques for measurement of human intestinal permeability

		Barrier Function			
Method	Tests Which Layer?	In vivo	In vitro	Neuroimmune Function	TJ Morphology
Cell monolayers (e.g., Caco-2/HT29)	Epithelium	−	+	−	−
Primary cell monolayers derived from organoids (human and animal model)	Epithelium	−	+	−	−
Ussing chambers + human mucosa	Epithelium	−	+	−	−
Human fecal or biopsy supernatant applied to animal mucosa	Epithelium	+	−	+/−	+/−
Zonula occludens-1 IHC	Epithelium	−	−	−	+
mRNA expression of TJ proteins	Epithelium	−	−	−	−
Urine excretion of oral probes	All barrier	+	−	−	−
Serum bacterial lipopolysaccharide and other biomarkers	All barrier	+	−	−	−
Duodenal mucosal impedance	Epithelium	+	−	−	−
Confocal endomicroscopy	Epithelium	+	−	+	−

IHC, immunohistochemistry; TJ, tight junction; +, the method demonstrates the function; −, the method is unable to demonstrate the function; +/−, borderline ability. [Adapted from Camilleri et al. (23).]

Autoradiography studies conducted in rat jejunal tissue using radiolabeled probes of different sizes showed that the upper portion of the villus allows flux of solutes with radius up to ∼6 Å, whereas the lower villus and crypt are permeable to solutes with radii of ∼10 Å and up to 60 Å, respectively (62), being >20 Å in the crypt in some reports (5). On the basis of in vitro studies conducted in cell lines, investigators have identified a pore pathway that is permeable to molecules with radii of ∼4 Å or less [typically ions such as Na^+^, K^+^, Cl^−^, and ${\text{HCO}}_{3}^{-}$ and possibly urea (NH_2_-CO-NH_2_)] and the leak pathway for flux of larger noncharged solutes (210) such as the probe molecules typically used in tests of intestinal permeability. Table 3 summarizes molecular sizes of probe molecules. From a biological perspective, the pore pathway is unlikely to accommodate passage of complex molecules such as bacterial toxins that may set up immune responses.

**Table 3.** Summary of molecular mass and diameter of probe molecules either published or estimated

		Molecular Diameter, Å	
Probe Molecule	Molecular Mass, Da	Reported	Estimated*
Urea	56	2.3	4.2
Erythritol	122	3.2	6.0
Rhamnose	164	8.2	6.9
Mannitol	182	6.7	7.2
Lactulose	342	9.5	9.7
Cellobiose	342	10.5	9.7
Sucralose	398	NA	10.4
PEG 400	194–634	NA	7.4–12.8
PEG 1,000	634–1,338	NA	12.8–18.1
Cr-EDTA	340	10.5	9.6
Dextran 4 kDa (e.g., FITC) and PEG 4,000	4,000	NA	30.0
PEG 10,000	10,000	NA	45.7
Bacterial endotoxins	10,000–20,000	NA	45.7–62.8
Lipopolysaccharides	50,000–100,000	NA	95.7–131.7
Dextran 40 kDa	40,000	NA	86.4
Dextran 70 kDa (e.g., rhodamine)	70,000	NA	111.8

Flux of molecules depends on the type of molecules and the type of defects in the intestinal barrier: Ions and water pass through tight junctions, antigens pass through apoptotic leaks, and macromolecules and bacteria pass through erosions, ulcers, or transcytosis (17). 1 Å = 0.1 nm. Cr-EDTA, chromium-labeled EDTA; NA, not available; PEG, polyethylene glycol.

* Calculated on the basis of the following formula: radius = 0.33 × (MM^0.46^), where MM is molecular mass.

Although it is claimed that monosaccharides such as mannitol or rhamnose traverse the epithelium via a paracellular “pore” pathway, and disaccharides such as lactulose via a “leak” pathway (150), examination of the molecular sizes (weight and diameter) suggests that the minor difference in molecular diameter may be associated with both monosaccharides and disaccharides traversing the epithelium via the same paracellular leak pathway. Transcellular passage occurs when there is a specialized, active transport mechanism, as occurs with glucose and fructose among saccharide molecules, but not with the probe saccharides used in testing intestinal permeability. Mucosal impedance and confocal endomicroscopic studies in humans demonstrate focal epithelial cell loss that may permit passage of larger molecules. Thus, Bischoff et al. proposed that flux of molecules depends on the type of molecules and the type of defects: Ions and water pass through tight junctions, antigens through apoptotic leaks, and macromolecules and bacteria through erosions, ulcers, or transcytosis (17).

There are major differences in in vivo measurements compared with in vitro measurements of barrier functions, as shown by the molecular size of probe molecules that can cross the epithelial barrier in humans in vivo, which is at least 10-fold smaller than in vitro. Thus, in healthy adults, ~0.1% of the mass of lactulose (molecular mass <500 Da) was recovered in urine after oral administration and passage through the entire small bowel and colon (172). Similarly, in childhood environmental enteropathy in Peru and Zambia, <1% of administered lactulose was recovered in urine (60). In contrast, molecules with molecular mass of ~4,000 Da [e.g., fluorescein isothiocyanate (FITC)-labeled dextran] easily traversed the 0.16–0.3 cm^2^ of human small intestinal mucosa in Ussing chamber in vitro (73).

Differences between in vivo and in vitro measurements may reflect additional functional barriers including the lamina propria, innervation (79, 82, 166) by submucosal neurons, and permeability of end-capillaries impeding passage of the probe molecules into the circulation in vivo. In addition, removing tissue from the body may reduce exposure to some factor that is involved in tissue integrity; indeed, most cell junctions in culture do not accurately reproduce the in vivo characteristics.

### Methods to Assess Intestinal Permeability

#### Recommended probe molecules to test intestinal permeability.

In vivo permeability measurements, derived from the analysis of urinary excretion of a single or two probe molecules (typically <400-Da molecular mass) after oral ingestion, are useful for within-subject comparison, as well as for comparisons between different groups. Urea and polyethylene glycol (PEG) are not recommended because of potential false positives as a result of low molecular mass (urea), potential bacterial metabolism (urea), or insufficient sensitivity (PEG; 17). Bacteria-related assays are also used to assess intestinal permeability. These include endotoxin [lipopolysaccharide (LPS)] assay; anti-LPS antibodies or d-lactate (bacterial lactate) in plasma or serum, respectively; butyrate or hemolysin in feces; quantification of bacteria in the inner layer of mucus in the colon on biopsies; and fat content of the liver by MRI or ultrasound. In general, these methods have limited standardization or lack of specificity or are invasive or expensive (17).

##### saccharides.

A combination of a monosaccharide, such as mannitol or rhamnose, and a disaccharide, such as lactulose or sucralose, has been reported to distinguish mucosal barrier function in inflammatory bowel disease from IBS (172). Nevertheless, the sensitivity is limited by the low levels of disaccharide excretion in urine within 2 h (~0.1%) and within 24 h (~0.5%) of ingestion (172). Lactulose (a disaccharide) and mannitol (a monosaccharide) are easily obtainable, are nontoxic, and are accurately measured using liquid chromatography‐tandem mass spectrometry (24) or electrochemical detection (58), thus allowing low doses to be used (e.g., 0.1 g mannitol and 1 g lactulose) and thereby avoiding a laxative effect or acceleration of transit, which could potentially confound the measurement. Inadvertent dietary consumption of [^12^C]mannitol in food, which would interfere with the test’s interpretation, has been resolved by either using rhamnose (not generally a food contaminant; 60) or replacing [^12^C]mannitol with [^13^C]mannitol (78). Measurements of each saccharide alone or the ratio of two saccharides such as lactulose and mannitol [lactulose-to-mannitol ratio (LMR)] or lactulose and rhamnose [lactulose-to-rhamnose ratio (LRR)] are commonly used. Nevertheless, further validation, including thorough characterization of test performance and reproducibility, is required. The ratio has the potential advantage of correcting for distribution or transit between individuals.

##### chromium-labeled ethylenediaminetetraacetic acid.

Chromium-labeled ethylenediaminetetraacetic acid (^51^Cr-EDTA) is a gamma-emitting isotope with a molecular mass of 339 Da and a 27-day half-life (17). Radiation from one dose of 0.12 mSv is at the level of background radiation. ^51^Cr-EDTA is administered orally in 10 ml of water; timed urine samples are gamma counted and expressed as percentage excreted. Unlike the saccharides, ^51^Cr-EDTA is not degraded by bacteria and, therefore, can measure both colonic and small intestinal permeability, based on the timing of the urine collection (17). In view of the gamma radiation, it is generally restricted to research studies; the sensitivity of this probe for noninflammatory diseases or effects of diet is not established.

##### polyethylene glycols.

PEG 400 is a mixture of 11 PEGs with molecular masses ranging from 194 to 634 Da; it has been used to assess altered intestinal permeability in small intestinal diseases such as celiac disease (28), pediatric and adult allergy, acute parasitic intestinal infections, and effects of NSAIDs (57, 95, 179, 199). However, an in-depth analysis of the diverse molecular entities showed that PEG molecules were excluded in both the high- and low-molecular weight range, possibly by a combined effect of the intestinal permeability barrier and an escape to compartments other than the urine (196). Moreover, recovery of PEG 400 in urine in 24 h varied with the relative molecular mass (*M*_r_) of each polymer from 25.9 to 68.5% (139). Larger PEG molecules with molecular weight 4,000 and 10,000 are typically used as nonabsorbable markers in intestinal perfusion studies; however, they have also been used in conditions such as alcoholic liver disease, which is associated with markedly increased permeability (158).

In summary, such probe molecule measurements are useful when timed collections correspond to the predominant location of the probe molecule within the bowel: predominantly the small intestine during the first 2 h, and almost exclusively the colon at 8–24 h, after ingestion (24, 172). These times of exposure apply almost equally in health and in stressed and disease states. However, PEG 400 has been used less frequently in recent years because of the variance in absorption and urinary excretion of the different-molecular weight entities requiring complex mathematical analysis (196).

##### examples of urine measurements of probe molecules to assess intestinal permeability.

Examples of urine measurements of probe molecules to assess intestinal permeability are discussed below under measurements to characterize normal structure and function of the intestinal barrier in human nutrition research, *Application of Intestinal Permeability Measurements in Humans*.

#### Serum biomarkers.

##### lipopolysaccharide.

Subclinical levels of serum LPS or endotoxin (the immunoadjuvant fraction of the outer cell membrane of gram-negative bacteria that is released on their lysis in the intestinal lumen) provide a marker of intestinal permeability. Impaired fasting blood glucose is associated with increased serum LPS and zonulin levels, which are independent and unrelated markers of increased intestinal permeability; the increased LPS may be biologically relevant as a trigger of in vivo platelet activation (27).

##### zonulin.

Zonulin, an ~47-kDa protein, regulates intestinal permeability reversibly by modulating intercellular tight junctions (59, 221); serum zonulin has been proposed as a marker of intestinal permeability (24, 182) and is strongly correlated with LMR in humans (122). Higher zonulin levels are also associated with higher waist circumference, diastolic blood pressure, and fasting glucose and increased risk of metabolic diseases (122, 151, 176), suggesting that bacterial endotoxins may play an important role in the development of the metabolic and vascular abnormalities commonly seen in obesity and diabetes-related diseases. In addition, a high-fat diet was associated with increased serum LPS (162).

##### intestinal fatty acid-binding proteins.

Intestinal fatty acid-binding proteins (I-FABPs) are small, unbound cytosolic proteins found mainly in enterocytes in the upper parts of small intestinal villi (161). They are sensitive markers for intestinal mucosal damage such as celiac disease (45). Serum FABP level has also been reported to increase during mild exertional heat stress or intestinal ischemia and reperfusion, though this was not always accompanied by a significant change in permeability as measured by LRR in urine or plasma, respectively (178, 184). Serum I-FABP was elevated in 5 of 20 patients with type 1 diabetes, though the mechanism and implications of this finding are unclear (84). Dietary factors such as gluten and casein supplementation in children with autism spectrum disorder did not alter urinary I-FABP (170).

##### examples of diet and stress effects on serum markers of intestinal permeability.

Measurements of serum markers of intestinal permeability appear promising in investigation of effects of diet on barrier function, as serum LPS levels increase after a high-fat meal in both healthy humans and humans with morbid obesity (33, 162). However, the sensitivity of serum LPS to detect effects of aspirin (a GI-damaging NSAID) is lower than 3-h urinary lactulose and LMR (77). On the other hand, there is correlation between serum levels of LPS and FABP and urine saccharide permeability measurement.

#### Endoscopic mucosal measurements.

These are invasive measurements that can be applied in research, typically in limited regions of the gut. Their application is relevant to small-scale clinical studies assessing stressful conditions or dietary components. Further studies and validation are required for both endoscopic-assisted methods.

#####  mucosal impedance measurement.

Mucosal impedance measurement (112) involves passing a 2-mm-diameter catheter through the endoscope for placement on the mucosa under direct visualization with decompressed lumen and all fluid aspirated. Initial studies were performed in the esophagus and have now been extended to intestine (53, 165). Through the catheter, with two 360° circumferential sensors, 2 mm apart, placed on the mucosa, a voltage transducer produces a 10-mA current at a frequency of 2 kHz. Impedance measurements are acquired in all quadrants of the duodenum. In the one study conducted to date, measurements of duodenal impedance in patients with IBS-constipation were not different compared with healthy controls, and these results confirmed the absence of effects on the basis of tissue studies in vitro (165); in addition, measurements of small intestinal mucosal impedance were not different in patients with eosinophilic esophagitis compared with healthy controls (225). Further studies on the effect of stress or diet are awaited.

##### confocal laser endomicroscopy.

Confocal laser endomicroscopy with 1-µm resolution enables visualization of cellular and subcellular structures in vivo in real time. After intravenous injection of 5 ml 10% fluorescein (which is actively transported into the lumen over ~30 min) and 40 mg methylscopolamine (anticholinergic to impede contractions), the laser is activated, and measurements are acquired before and after exposure to food. In patients with IBS with suspected food intolerance, exposure to candidate food antigens caused immediate breaks, increased intervillous spaces, and increased intraepithelial lymphocytes in the intestinal mucosa (65). These changes responded to exclusion diets and correlated with clinical improvement. In the terminal ileal epithelium, median epithelial gap densities for control patients and patients with IBS were 6 and 32 gaps per 1,000 cells, respectively (*P* < 0.001), with numerically higher gap density in female and younger patients (207).

#### General pitfall with all methods of measurement of intestinal permeability.

A general pitfall applicable to all these methods is a lack of standardization of the method (including probe molecules or serum biomarkers, urine collection, and assay methods), a lack of robust normal data (including consideration of age, sex, BMI, circadian rhythm, and standardization of diet during at least the 24 h of collection of biological samples), and performance characteristics of the test including validity based on responsiveness to perturbations or treatments. In summary, at present, it is unclear what constitutes “normal” values for the diverse measurements, and each article in the literature has to assess the “altered” state (e.g., disease, treatment, or nutrient) with a healthy or placebo control.

### Application of Intestinal Permeability Measurements in Humans

#### Illustrations of functioning mucosal barrier in noninflammatory gut conditions.

Table 4 (8, 15, 37, 41, 51, 53, 65, 74, 113, 128, 130, 137, 145, 157, 165, 172, 180, 190, 194, 205, 207, 214, 219, 237, 238) and Table 5 (16, 73, 123, 165, 167, 217, 218, 232) summarize the application of in vivo and in vitro measurements of intestinal permeability in IBS, which was selected because it is not associated with overt mucosal defects or inflammation and is more likely to reflect the magnitude of changes in permeability that might result from ingested foods or other substances. These data are, therefore, the most representative of what might occur in the general population or under conditions of stress, to provide a basis for proposing diet studies.

**Table 4.** Summary of in vivo measurements of intestinal permeability in humans, focusing on studies that include noninflammatory disease

Reference	Year	Method	Patients with IBS and Controls, *n*	IP of Patients with IBS, % above normal or LMR	Comments
Strobel et al. (194)	1984	C/M	15 IBS and 10 controls	Mean ratio: 0.024 (normal 0.037)	Nonbiopsied volunteers as controls
Lobley et al. (130)	1990	Raffinose/l-arabinose	62 IBS and 40 controls	Mean ratio: 0.016 (normal 0.015)	No significant difference in IP between IBS and controls
Barau and Dupont (8)	1990	L/M	17 IBS and 39 controls (children)	47 vs. 0% above normal for IBS vs. controls, respectively (normal <0.0245)	Threshold of normal defined by a control group of children without IBS
Vogelsang et al. (219)	1995	L/M	40 symptomatic and 30 controls	30% of symptomatic patients above normal (>0.030)	Patients with “nonspecific” GI symptoms
Dainese et al. (37)	1999	L/M	33 IBS and 0 controls	12% IBS above normal (>0.025)	IP normal in 88% of subjects
Berstad et al. (15)	2000	^51^Cr-EDTA	18 IBS and 0 controls	Excretion: 0.07% in IBS	Patients with IBS (abdominal pain and/or diarrhea) used as controls in IBD study
Spiller et al. (190)	2000	L/M	10 PI-IBS, 21 acute *Campylobacter* enteritis, and 12 controls	50% IBS vs. 12 controls; mean LMR: 0.060; range: 0.008–0.22 (normal <0.03)	Increased IP in subset of patients with PI-IBS compared with asymptomatic controls
Tibble et al. (205)	2002	L/R	339 IBS and 263 organic disease	Mean ratio: 0.028; range: 0.005–0.216 (normal <0.05)	Permeability of small intestine close to normal in IBS
Marshall et al. (137)	2004	L/M	132 IBS and 86 controls	35.6 vs. 18.6% above normal for IBS vs. controls, respectively (>0.020 LMR)	After outbreak of acute gastroenteritis, SB IP was slightly elevated in IBS (no difference between PI-IBS and non-PI-IBS)
Dunlop et al. (51)	2006	^51^Cr-EDTA	15 IBS-D + 15 IBS-C with 15 controls and 15 PI-IBS + 15 non-PI-IBS with 12 controls	Excretion: in proximal SB: 0.19% IBS-D, 0.085% IBS-C, 0.07% controls; in SB: 0.43% PI-IBS, 0.84% non-PI-IBS, 0.27% controls	There were 2 studies: 1 comparing IBS-D and IBS-C vs. controls and 1 comparing PI-IBS and non-PI-IBS with IBS-D vs. controls; there may be subtle differences in IP between IBS subgroups
Shulman et al. (180)	2008	L/M and S/L	109 Children with IBS or functional abdominal pain and 66 controls	Increased SB and colonic permeability	No correlation between GI permeability and pain-related symptom or stool form
Park et al. (157)	2009	PEG 3,350-to-PEG 400 ratio by HPLC	38 IBS (all subtypes) and 12 healthy controls	Increased in whole IBS group	No relationship of increased permeability and positive L breath test
Zhou et al. (238)	2009	L/M	54 IBS-D and 22 controls	Increased LMR in 39% of patients	Relationship to increased abdominal pain and visceral and thermal sensitivity
Kerckhoffs et al. (113)	2010	PEG	14 IBS (all subtypes) and 15 healthy controls	No difference between IBS and healthy controls	NSAIDs increase permeability more in IBS than in healthy controls
Zhou et al. (237)	2010	L/M	19 IBS-D and 10 controls	Increased in 42% of patients	
Rao et al. (172)	2011	L/M	12 IBS-D, 12 healthy, and 10 inactive or treated UC or microscopic colitis	Increased urine M excretion at 0–2 and 2–8 h and L excretion at 8–24 h in IBS-D	Demonstrated validity of individual sugar excretion as well as LMR
Gecse et al. (74)	2012	^51^Cr-EDTA	18 IBS-D, 12 IBS-C, 13 inactive UC, and 10 healthy	Decreased in proximal small intestine of IBS-C; increased in colon of IBS-D	Elevated gut permeability is localized to the colon both in IBS-D and in inactive UC
Vazquez-Roque et al. (214)	2013	L/M	45 IBS-D: trial of ±gluten diets	GCD increased SB permeability (based on M and LMR); no increase in colon permeability	GCD significantly decreased expression of ZO-1, claudin-1, and occludin in rectosigmoid mucosa; all effects of gluten were greater in patients positive for HLA DQ2/8
Del Valle-Pinero et al. (41)	2013	4 probes: S, sucrose, M, and L	20 IBS and 39 matched healthy controls	Colonic permeability significantly lower in IBS compared with healthy controls, shown by lower S excretion in IBS compared with controls	IBS subgroups not specified
Turcotte et al. (207)	2013	Confocal laser endomicroscopy	16 IBS and 18 healthy controls	Median epithelial gap densities for controls and IBS were 6 and 32 gaps per 1,000 epithelial cells, respectively	Median difference in gap density between IBS and controls was 26 (95% CI: 12–39) gaps per 1,000 cells; small effects of age and sex
Fritscher-Ravens et al. (65)	2014	Confocal laser endomicroscopy	36 IBS with suspected food intolerance	No overall differences, but positive results in 22 of 36 patients: increased number of IELs, formation of epithelial leaks/gaps, and intervillous spaces widened	Diluted food antigens administered directly to the duodenal mucosa; however, no correlation with conventional histology
Mujagic et al. (145)	2014	Sucrose excretion and LRR in 0–5-h urine; 0–24- and 5–24-h S-to-erythritol ratio	34 IBS-D, 21 IBS-C, 30 IBS-M, 6 IBS-U, and 94 healthy controls	The 0–5-h LRR only different in IBS-D vs. healthy controls; no other differences in gastroduodenal or colonic permeability	Analysis adjusted for age, sex, BMI, anxiety or depression, smoking, alcohol intake, and use of medication
Peters et al. (165)	2017	L/^13^C-M, mucosal impedance, and serum LPS	19 IBS-C and 18 healthy volunteers	Normal SB and colonic permeability in IBS-C	Concordant results (normal) using duodenal mucosal impedance, ex vivo barrier measurements, and colonic mucosal expression of occludin, ZO-1, 2, and 3, and claudin genes
Edogawa et al. (53)	2018	L/^13^C-M	9 healthy volunteers	Increased L SB permeability by indomethacin, recovered to baseline 4–6 wk later	Only women demonstrated decreased fecal microbial diversity, including an increase in *Prevotella* abundance, after indomethacin
Linsalata et al. (128)	2018	urinary sucrose, L, and M over 5 h and circulating biomarkers	39 IBS-D and 20 healthy volunteers	There were 2 distinct IBS-D subtypes identified, 1 with increased L, sucrose excretion, and I-FABP and DAO levels, suggesting increased permeability of small intestine	Inflammatory parameters and markers of bacterial translocation (IL-6 and LPS) were significantly higher in IBS-D with increased permeability of small intestine

Here, *n* = no. of subjects. BMI, body mass index; C, cellobiose; CI, confidence interval; ^13^C-M, [^13^C]mannitol; ^51^Cr-EDTA, chromium-labeled EDTA; DAO, diamine oxidase; GCD, gluten-containing diet; GI, gastrointestinal; HLA DQ2/8, human leukocyte antigen DQ2 or DQ8; IBD, inflammatory bowel disease; IBS, irritable bowel syndrome; IBS-C, IBS with constipation; IBS-D, IBS with diarrhea; IBS-M, IBS with mixed bowel habits; IBS-U, unsubtyped IBS; IELs, intraepithelial lymphocytes; I-FABP, intestinal fatty acid-binding protein; IP, intestinal permeability; L, lactulose; LMR, lactulose-to-mannitol ratio; LRR, lactulose-to-rhamnose ratio; M, mannitol; PEG, polyethylene glycol; PI-IBS, postinfectious IBS; R, rhamnose; S, sucralose; SB, small bowel; UC, ulcerative colitis; ZO-1, zonula occludens-1.

**Table 5.** In vitro effects of soluble factors on barrier function and tissue expression in studies including noninflammatory disease

Reference	Year	Method	IBS Group, *n*	Permeability	Comments
Gecse et al. (73)	2008	FSN applied to murine colonic strips mounted in Ussing chambers; FITC-dextran transfer	52 All IBS subtypes and 25 controls	Increased with IBS-D supernatants, no difference with IBS-C	FSN also rapidly increased phosphorylation of myosin light chain and delayed redistribution of ZO-1 in colonocytes
Piche et al. (167)	2009	Colonic biopsies mounted in Ussing chambers; fluorescein-5-(and-6)-sulfonic acid as probe and ZO-1 and occludin expression	51 IBS, all subtypes, and 14 controls	Increased FITC paracellular permeability in all IBS subtypes; reduced ZO-1 expression	No difference in occludin expression; increase in FITC-dextran in Caco-2 cell monolayer, which correlated with abdominal pain score
Lee et al. (123)	2010	Colonic biopsies in Ussing chambers; horseradish peroxidase as probe	20 IBS-D and 30 controls	Increased in IBS-D compared with controls	Increased permeability decreased with the mast cell tryptase inhibitor nafamostat
Bertiaux-Vandaële et al. (16)	2011	Colonic mucosal biopsies and ZO-1, occludin, and claudin-1 expression	50 IBS (-C, -D, -A, or -U) and 31 controls	Occludin and claudin-1 expression decreased in IBS-D but not in IBS-C/A	Occludin (*r* = 0.40) and claudin-1 (*r* = 0.46) expression significantly correlated with duration of symptoms
Vivinus-Nébot et al. (217)	2012	Colonic biopsies mounted in Ussing chambers; fluorescein-5-(and-6)-sulfonic acid	34 IBS, all subtypes, and 15 controls	Increased in all IBS subtypes	Also higher number of mast cells, and spontaneous release of tryptase; worse in IBS with allergic factors
Vivinus-Nébot et al. (218)	2014	Cecal biopsies: Ussing chambers, FITC-sulfonic acid as probe, and mRNA expression of TJ proteins (ZO-1, α-catenin, and occludin)	49 inactive IBD (IBS), 51 IBS, and 27 controls	Increased permeability and lower expression of ZO-1 and α-catenin in both inactive IBD and IBS	Persistent increase in TNF-α in colonic mucosa may contribute to the epithelial barrier defects in quiescent (inactive) IBD but not in IBS
Peters et al. (165)	2017	TMR and FITC-dextran flux (4 kDa)	19 IBS-C and 18 controls	No differences	Results consistent with in vivo permeability measurements
Wu et al. (232)	2017	H&E and semiquantitative immunohistochemistry for phosphorylated MLC, MLC kinase, and claudins-2, -8, and -15	27 IBS-D ±gluten diet	Increased MLC phosphorylation and colonocyte expression of the paracellular Na^+^ channel claudin-15 by GCD	Small intestine MLC phosphorylation increased by GCD correlated with increased intestinal permeability

Here, *n* = no. of subjects. FITC, fluorescein isothiocyanate; FSN, fecal supernatant; GCD, gluten-containing diet; H&E, hematoxylin-eosin; IBD, inflammatory bowel disease; IBS, irritable bowel syndrome; IBS-A, IBS with alternating constipation and diarrhea; IBS-C, IBS with constipation; IBS-D, IBS with diarrhea; IBS-U, unsubtyped IBS; MLC, myosin II regulatory light chain; TJ, tight junction; TMR, transmucosal resistance; ZO-1, zonula occludens-1.

Although there is mention of leaky gut in various conditions, from allergies to chronic fatigue syndromes, it is important to note that the evidence is often based on serum IgA and IgM responses directed against commensal bacteria, suggesting bacterial translocation across the intestinal barrier. On the other hand, permeability probes of lower molecular weight than bacteria show perturbations in barrier function such as those due to NSAIDs (20), and their effect is reversed with lubiprostone (36, 111). These intriguing results require replication. Thus, it is conceivable that under stressful or dietary stimuli, there may be alterations in mucosal permeability in the healthy state in addition to the presence of circulating immunoglobulins against commensal bacteria that might reflect the effects of a pathological state.

#### Application of oral probes to measure barrier changes among healthy subjects exposed to different diets.

In healthy subjects, there are two relevant scenarios. First, normal gut barrier function can be exposed to diets or dietary components to determine whether permeability is increased. For example, when fasting, normal subjects were given 300-cal drinks of either glucose, saturated fat such as cream, orange juice, or only water; only cream caused an increase in LPS concentration (suggesting increased permeability) and Toll-like receptor 4 expression indicative of cellular inflammation (44). Coadministration of fiber reversed the effects of the high-fat, high-calorie meal (76).

#### Dysfunction of gut mucosal barrier due to stressors and effects of nondrug interventions.

The normal gut barrier function can be stressed so that diets or dietary components can be assessed for their role in restoring permeability to baseline normal for that individual. Examples of such stressors resulting in mucosal perturbation are exposure to NSAIDs (18, 144, 181) or endurance exercise with ischemia-induced changes in permeability. Although the effects of NSAIDs on permeability are reversible pharmacologically with lubiprostone (36, 111) and demonstrate that prostaglandins may restore normal barrier function, the examples in Table 6 (18, 103, 109, 119, 131, 144, 168, 181, 213) illustrate the efficacy of substances in the diet to reverse these effects.

**Table 6.** Examples of stressors altering permeability in health and reversal by dietary intervention

		Effects on Barrier Function					
Barrier Stressor and Clinical Scenario	Specific Study	Intestinal permeability	TJ	Mucosal damage	Other effects	Dietary Intervention	Reference(s)
Endurance exercise; marathon runners with fecal occult blood or bloody diarrhea	Biking challenge	Urine iohexol (MM 821 Da) ↑	ND	Serum I-FABP ↑, zonulin ↓	ND	Noninterventional study	(109)
	Running challenge	LRR ↑ and correlated with core temperature (e.g., >39°C)	ND	ND	ND	ND	(168)
	Biking challenge	LRR not different with citrulline Rx	ND	Serum I-FABP ↑ reversed with citrulline	Gastric hypoperfusion reversed with citrulline	Citrulline vs. alanine	(213)
	Biking challenge	ND	ND	Serum I-FABP ↑ reversed with sucrose	Gastric hypoperfusion not different with sucrose	Sucrose vs. nitrate	(103)
NSAID enteropathy; NSAIDs cause small bowel ulcers and inflammation	Diverse NSAIDs including indomethacin	^51^Cr-EDTA, saccharides	ND	ND	ND	Noninterventional studies	(18, 144, 181)
	Indomethacin	LRR reduced with zinc carnosine	ND	ND	HT29 cell proliferation ↑ with zinc carnosine vs. ZnSO_4_	Zinc carnosine vs. placebo	(131)
	Aspirin	*Bifidobacterium lactis* BB-12 and GOS reduce colonic permeability: urine SLR and sucralose	ND	ND		*Bifidobacterium* strains (*B. lactis* BB-12 and *B. adolescentis* IVS-1) and GOS prebiotic	(119)

51 Cr-EDTA, chromium-labeled EDTA; GOS, galactooligosaccharide; I-FABP, intestinal fatty acid-binding protein; LRR, lactulose-to-rhamnose ratio; MM, molecular mass; ND, not determined; Rx, prescription; SLR, sucralose-to-lactulose ratio; TJ, tight junction expression; ↑, increase; ↓, decrease.

#### Illustration of dietary activation of local protective immune or barrier mechanisms in disease states.

Although not the main focus of this article, it is intriguing that indigo naturalis, through the aryl hydrocarbon receptor (AhR), was able to stimulate the protective IL-22 and resulted in mucosal healing in a randomized, controlled trial in patients with ulcerative colitis (146). Other examples pertaining to dietary interventions in severe burns, nutritional depletion, and Crohn’s disease are shown in Table 7 (11, 90, 142, 163, 239).

**Table 7.** Examples from the literature of in vivo human studies showing alterations in intestinal permeability as a result of gut-directed therapy

Reference	Year	Therapy Studied	Comments
Hulsewé et al. (90)	2004	Glutamine intravenous	Patients with nutritional depletion and increased IP did not improve after glutamine-enriched parenteral nutrition
Zhou et al. (239)	2003	Glutamine enteral	Patients with 50–80% burns: urinary LMR in enteral glutamine group was lower than standard enteral formula
Peng et al. (163)	2004	Glutamine enteral	Patients with 30–75% burns: plasma DAO activity and urinary LMR in enteral glutamine group were lower than in untreated burn group
Benjamin et al. (11)	2012	Glutamine	In patients with Crohn’s disease, glutamine and active control (whey) both reduced LMR
Meng et al. (142)	2011	Rhubarb	Patients *day 3* postburns: plasma DAO activity in rhubarb-treated group was lower than in controls

DAO, diamine oxidase; IP, intestinal permeability; LMR, lactulose-to-mannitol ratio.

### Conclusions on Testing of Intestinal Permeability

Normal values vary for each probe molecule, and further validations of normal values are required. There is lower intraindividual coefficient of variation compared with interindividual variation. Hence, intraindividual comparisons of diets or other perturbations appear to be preferable in humans when using urinary recoveries after oral administration. However, with careful test performance, individual saccharide excretion and ratio of disaccharide to monosaccharide excretion provide valid information on intestinal permeability in noninflammatory conditions and, therefore, could potentially serve as useful probes to study effects of diet on either increasing permeability or restoring to normal after a stressor.

## POTENTIAL OF DIETARY AND NONDIETARY FACTORS TO IMPACT COMPONENTS OF THE INTESTINAL MUCOSAL BARRIER

Maintenance of a healthy gut barrier requires the functional integration and homeostasis of the gut microbiota, mucus production, intestinal epithelial cells, and the mucosal immune system. Each of these components is continually responding to cues from the other components, as well as to signals derived from the intestinal lumen. Food-derived compounds and their metabolites have broad immunomodulatory and physiological effects and can act to modulate cellular and barrier function by direct interactions with immune and epithelial cells and indirectly by altering gut microbial composition and/or function (39, 231). The gut microbiota, including archaea, eukaryotes, bacteria, and viruses, can reach densities exceeding 10^11^ cells per gram in the colon (118). An altered composition of the gut microbiota has been documented in diverse human diseases, and a causal role of the gut microbiota in metabolic and inflammatory diseases has been demonstrated repeatedly in animal models (186, 188). Many dietary compounds serve as chemical precursors that feed into a complex network of still incompletely understood microbial and host metabolic pathways (50). The small-molecule metabolites that result from these pathways can be absorbed into the bloodstream and circulate throughout the body and may interact with tissues and be cometabolized into additional chemical forms or eventually excreted (148).

### Dietary Compounds and Metabolites

Dietary metabolites can interact directly with host cells via surface transmembrane G protein-coupled receptors (GPCRs) or through transcription factors such as the AhR. Metabolites can also have transcriptional and epigenetic effects via acetylation and deacetylation of histone proteins. Dietary metabolites can thus elicit immediate responses through altering cellular signaling and also have long-term effects through epigenetic modifications (200). GPCRs interact with food-derived metabolites such as short-, medium-, and long-chain fatty acids, as well as products of other metabolic pathways such as those involved in tryptophan (Trp) metabolism. GPCRs have a major role in promoting anti-inflammatory responses, inducing immune tolerance, and controlling metabolic function. The AhR is a cytosolic ligand-activated transcription factor expressed by intestinal epithelial cells and most immune cells (193). AhR can be activated and/or inhibited by numerous dietary molecules including flavonoids and carotenoids. AhR activity is critical for T helper type 22 (Th22) and Th17 cell activation and IL-22 production (234). IL-22 has a critical role in barrier defense through enhancing epithelial regeneration, increasing mucus production, stimulating wound healing, reinforcing epithelial tight junctions, and enhancing production of AMPs (56). A lack of IL-22 has been linked to host pathologies including infections and metabolic disorders (222, 227). Some evidence from randomized, controlled trials and epidemiological studies suggests that consumption of flavonoid- and carotenoid-rich foods may be beneficial for maintaining metabolic and cardiovascular health (223), but whether this is due to enhanced signaling through AhR or through effects on gut barrier function is not known (72).

#### Dietary fiber and barrier function.

It is notable that the only present evidence for a dietary intervention that may help to fortify intestinal barrier function in healthy humans, from a conceptual standpoint, is the consumption of dietary fiber. Microbiota-accessible carbohydrates (MACs) found within dietary fiber (187) are complex carbohydrates that are indigestible by host enzymes but can be fermented by gut microbiota primarily in the colon. The association between consumption of dietary MACs and maintenance of a functional intestinal barrier (132) can be, at least partly, attributed to the production of short-chain fatty acids (SCFAs) in the gut lumen. SCFAs support intestinal epithelial cell proliferation (93, 115, 175, 197) and protect intestinal barrier integrity (31, 67, 191) via a combination of mechanisms. They constitute the primary energy source for the colonocytes, induce mucus secretion, alter epigenetic processes by inhibiting histone deacetylase, and activate free fatty acid receptors 2 and 3 and GPCRs on epithelial cells (25, 42, 116).

The importance of mucus integrity to host health is evidenced by spontaneous colitis that develops in mice when components of the intestinal mucus have been genetically compromised (13, 66, 228). Many bacterial members of the gut microbiota have the capacity to degrade and consume portions of the mucus layer, but in a healthy gut, microbes are excluded from the tight, inner layer of mucus closest to the epithelium (101). Highly controlled experiments in animal models have revealed that mucus consumption by gut microbes increases when dietary fiber becomes scarce in the diet, and mucus glycans appear to serve an important role as a backup food source for many fiber-degrading microbes (138, 189, 198). As microbial consumption of host-secreted mucus glycans increases in fiber-scarce dietary conditions, the mucus layers become thinner, as determined through quantitative measurements of the mucus layer in mouse models (46, 52). Furthermore, markers of mucosal and systemic inflammation increase concomitantly with the low fiber-induced mucosal thinning (46, 52). These diet-driven modifications of the mucus layer and induction of inflammation are consistent with findings in mouse colitis models and in humans with inflammatory bowel diseases; microbial encroachment of the inner mucus layer is linked to intestinal inflammatory states (100). Similarly, a high-fat diet (60% of energy) induced microbiota encroachment and low-grade inflammation in mice that was ameliorated with the supplementation of the dietary MAC inulin (20% wt/wt; 241). Together, these findings reveal that aspects of the Western diet and, specifically, low dietary fiber content, alter the relationship between the human gut microbiota and the gut mucus layer and may predispose those ingesting a fiber-poor diet to increased intestinal inflammation.

Dietary fiber leading to the production of SCFAs can also help regulate the intestinal mucosal immune barrier. SCFAs modulate the size and function of the T cell network in the gut through G protein-coupled receptor 43 (GPR43)-dependent mechanisms and through histone deacetylase inhibition (4, 68, 104, 105, 183). Disruption of the homeostasis of regulatory T cells (which regulate Th cells) results in the loss of immune tolerance and development of aberrant effector responses, in particular Th17 cells (10). Th17 cells have been implicated in the development of inflammatory bowel disease (71).

Administration of MAC to healthy animals (143) or animals with a compromised gut barrier (91, 92) consistently showed improved colonic barrier function as evidenced by increased expression of colonic tight junction proteins. Other interventions with SCFAs have also yielded positive results on intestinal permeability (61, 89). However, in healthy subjects, only one study reported improved small intestinal permeability after consumption of inulin-enriched pasta (11.0 g/day of fructans) for 8 wk compared with control pasta (1.4 g/day of fructans; 174). In contrast, intestinal permeability was not affected by synbiotic or prebiotic supplementation in healthy men (202, 229, 230) or men with well-controlled type 2 diabetes (160). The dose of oligosaccharide (fructooligosaccharide or galactooligosaccharide) in these studies varied between 100 mg/day and 20 g/day.

#### Amino acids, proteins, and barrier function.

One paradigm by which a dietary nutrient plays a role in the regulation of intestinal barrier immune function is through Trp, an essential amino acid that must be supplied exogenously in the diet. Trp can be directly transformed into indoles and derivatives or enter the kynurenine (Kyn) pathway in immune and epithelial cells via indoleamine 2,3-dioxygenase 1 (IDO1). Trp can also pass through the serotonin (5-hydroxytryptamine pathway) via Trp hydroxylase (49, 70, 96, 121). The integrity of the barrier in a mouse model can be directly affected by metabolites produced by gut microbes such as indole-3-propionic acid (IPA), a microbial metabolite of the amino acid Trp that binds to the pregnane X receptor and influences immunity and barrier integrity (49, 216). Alterations in Trp metabolism have been linked with several human diseases (2, 72). Genetic susceptibility to inflammatory bowel disease due to deletion of the caspase recruitment domain family member 9 (*Card9*) gene is associated with an altered microbiota that has an impaired capacity to metabolize Trp to AhR ligands (121).

Kyn is an AhR agonist generated from Trp by IDO1 and tryptophan 2,3-dioxygenase (TDO). An increased activity of IDO1 and increased serum Kyn levels are seen in individuals with metabolic syndrome, and correlations between the Kyn-to-Trp ratio and obesity, metabolic syndrome, BMI, and blood triglycerides have been described (133). Patients with type 2 diabetes have been shown to have increased gut permeability associated with metabolic endotoxemia (86). Bariatric surgery improves metabolic disease and also reduces gut permeability in conjunction with altering gut microbiota and increasing levels of IPA (96, 206). Experimental studies have shown that bacterial-derived IPA reduces intestinal permeability, suggesting a role for Trp metabolites in the control of metabolism and gut permeability (96).

Circulating concentrations of 4-ethylphenylsulfate (EPS), a microbial-host cometabolite of dietary tyrosine, were 46-fold elevated in a mouse model of autism, where the animals had reduced gut barrier function. Oral treatment with *Bacteroides fragilis* (10^10^ colony-forming units/48 h) to improve barrier function completely normalized EPS concentrations and improved autism-like behavior (88).

There is some epidemiological evidence showing an increased risk of developing inflammatory bowel disease in subjects with a high protein intake, specifically with meat protein [reviewed by Kakodkar and Mutlu (107)]. A mechanism that may help explain this association lies in the interactions between high protein levels reaching the colonic microbiota and the fermentation by these microbes, which releases toxic compounds such as ammonia, phenols, branched-chain amino acids, and hydrogen sulfide (127, 233). An elegant study examining effects of altering diet composition on intestinal permeability and colitis development in mice clearly demonstrated that increasing protein in the diet increased gut permeability and also the severity of colitis in a microbiota-dependent and microbiota-independent manner (129).

#### Sugar consumption and barrier function.

Western diets high in fat and sugars have been associated with impaired intestinal barrier function in animal models (81, 220). Intestinal glucose absorption occurs primarily through sodium/glucose cotransporter 1 (SGLT1) but can also occur through tight junctions at very high intraluminal glucose concentrations and a sufficient osmotic gradient to promote volume flow (7). However, given the very rapid absorption of monosaccharides in the proximal 70 cm of the small intestine (21, 99), it is unclear whether intraluminal concentrations of glucose are sufficient to alter intestinal permeability. However, Thaiss et al. recently demonstrated in an elegant set of in vitro and mouse experiments that hyperglycemia, even in the absence of obesity, could increase gut permeability via alterations in tight junction and adherens junction integrity (203). Another study in middle-aged human subjects showed that those individuals with dysglycemia had encroachment of bacteria into the normally sterile inner colonic mucus layer, representing a loss of barrier function (30).

In a mouse model, high levels of dietary fructose in excess of glucose have also been shown to increase gut permeability and allow for enhanced translocation of LPS due to effects on tight junctions, reduced mucus thickness, and a reduced expression of antimicrobial proteins (220). A link between fructose consumption and the development of metabolic syndrome and/or nonalcoholic fatty liver disease in humans has been demonstrated in several human studies [154, 164, 191; reviewed by Jensen et al. (97)]. It has been suggested that consumption of high levels of dietary fructose increases systemic endotoxin levels by increasing intestinal permeability through both direct and indirect methods (98, 204). These include the effects of fructose metabolism within gut enterocytes and hepatocytes. In hepatocytes, phosphorylation of fructose can result in transient intracellular phosphate and ATP depletion (9, 35). This, in turn, causes a transient reduction in protein synthesis, mitochondrial dysfunction, an increase in uric acid production, and resultant enhanced systemic oxidative stress that can then increase gut permeability [reviewed by Zhang et al. (236)]. A similar mechanism has been proposed to directly occur in gut epithelial cells. Fructose is absorbed through glucose transporter 5 (GLUT5) and GLUT2-mediated facilitative diffusion. Given that dietary fructose is often associated with glucose in humans, the active fructose uptake through GLUT2 reduces the luminal concentration of fructose; therefore, the potential effects of dietary fructose may be different from those observed in experimental animals fed fructose alone. Therefore, the potential effects of dietary fructose may be different from those observed in animal and human experimental models fed fructose relative to glucose at levels not typically consumed in the human diet. Studies in mice have demonstrated that increased dietary fructose results in a decrease in occludin and zonula occludens-1 (ZO-1) gene expression in small intestinal epithelium, suggesting increased permeability (102). Indeed, these changes were not seen in fructokinase A/C knockout mice, indicating that the changes in epithelial tight junctions were dependent on fructose metabolism through fructokinase.

#### High-fat diets and bile acids.

The fat content of the diet has a significant influence on bile acid secretion. In addition to their role in dietary lipid absorption and cholesterol homeostasis, bile acids act as signaling molecules via two major signaling pathways: G protein-coupled bile acid receptor (GPBAR1, or TGR5) and members of the nuclear hormone receptor superfamily including the farnesoid X receptor (FXR; 63). Animal studies indicate that both TGR5 and FXR contribute to the integrity of the intestinal barrier (32, 48, 69, 94). Alternatively, some bile acids exert direct, receptor-independent toxicity toward intestinal epithelial cells (34), based on the detergent properties of bile acids (149), induction of apoptosis (3), and induction of changes in tight junction proteins (171). Finally, some unconjugated bile acids may provoke colonic mucus secretion, probably through a direct action on mucus-secreting cells.

High-fat feeding and bile acids also induce intestinal inflammation and alter the gut microbiota composition (40, 159). Levels of *Akkermansia muciniphila* were 100-fold lower in mice fed a high-fat diet compared with mice on a control diet (55), and gut barrier dysfunction in these animals was reduced by administration of *A. muciniphila* for 4 wk, probably by stimulating the growth of the intestinal mucus layer. Other studies have linked high-fat diets to increases in sulfate-reducing bacteria and barrier dysfunction through the inhibition of butyrate oxidation in colonocytes (6, 26, 43, 173, 235). In animal models, it has been shown that fat from milk increases the secretion of taurocholic acid in bile, which serves as a substrate for *Bilophila wadsworthia*, another hydrogen sulfide-producing species disrupting barrier function (47).

#### Emulsifiers and surfactants.

Emulsifiers are detergent-like molecules that are used extensively in processed foods. Studies in mice have shown that two commonly used emulsifiers, carboxymethylcellulose and polysorbate-80, induced metabolic syndrome and low-grade inflammation in conjunction with gut barrier dysfunction (29). Other surfactants that are used in the food industry such as monoglycerides, lecithins, glycolipids, fatty alcohols, fatty acids, and organic solvents used in food and beverage preparation (117) disrupt the intestinal barrier in animal models (80), as well as opening tight junctions in cell culture (124).

Although these observations suggest putative roles for these agents, it is unclear whether the mass ingested by humans is sufficient to be biologically or clinically relevant, particularly as they are diluted by the ~2 liters of aqueous solutions entering the GI tract with every meal and because of sequestration of fats in micelles and their ultimate absorption, preventing direct contact with the epithelium to exert their surface-active properties.

#### Alcohol.

Both acute and long-term intake of alcohol consistently increased small intestinal epithelial barrier permeability (19, 192, 224), but no data are available on the effects of ethanol on the human colonic barrier. Although the exact mechanism of increased permeability remains largely unknown, direct damage to epithelial cells, changes in the expression of the tight junction-associated and adherens junction proteins, and changes in the intestinal microbiota have been shown to be involved (54). Gut permeability to PEG 1,500, 4,000, and 10,000 is increased in people with alcohol use disorder, allowing large macromolecules through the intestinal barrier, and plasma LPS (endotoxin) levels increased in parallel with increases in gut permeability (158). There is also evidence of a marked increase in lactulose absorption as well as in urinary LMR in people with alcoholism and chronic liver disease compared with people with alcoholism and no liver disease and nonalcoholics with liver disease (114).

#### Nutrients in combination.

One example of effects of nutrients in combination is illustrated by a randomized, double-blind, placebo-controlled trial in 120 children, aged 2 mo to 9 yr, from an urban shanty community, that showed that glutamine alone or glutamine plus vitamin A and zinc (both gut-trophic nutrients) reduced intestinal permeability (%lactulose excretion and LMR) compared with placebo (zinc plus vitamin A vehicle; 126). Beneficial effects of other gut-trophic nutrients (such as arginine, dietary fiber, glutamine, glutathione, SCFAs, vitamin A, and zinc) on intestinal growth, adaptation, and barrier function have also been described. These are reviewed elsewhere (240).

### Diet-Independent Factors Affecting Intestinal Permeability

Diet is an important, but not the only, factor that affects the intestinal barrier. Other lifestyle-associated factors, including stress, obesity (BMI), aging, smoking habits, and use of medication, should at least be reported in human diet-gut barrier studies and eventually be considered as confounding factors in statistical analysis. Unfortunately, most studies generally do not control for dietary intake. Therefore, it cannot be excluded that differences in barrier function between target groups and controls are due to differences in dietary intake rather than the lifestyle factors under consideration. Additionally, it is conceivable that studies focused on healthy humans, where diet-independent factors that have an influence on the intestinal epithelial barrier (described below), could be used to test dietary-based strategies. Such studies could fortify intestinal barrier function as a method to maintain or restore health and reduce risk of disease.

#### Stress.

Limited data are available on the impact of psychological stress on barrier function in humans. Acute stress induced by a public speech test in healthy subjects increased intestinal permeability versus control conditions in an LMR test (0.059 ± 0.040 vs. 0.030 ± 0.022, mean ± SE; *P* < 0.01) in a subgroup of subjects that exhibited increased salivary cortisol. Exogenous corticotropin-releasing hormone mimicked these effects, whereas prior administration of a mast cell stabilizer abolished them (211). Strenuous exercise is believed to induce a combination of physical and psychological stress. In addition to the effect mentioned above, exercise leads to redistribution of blood away from the splanchnic area, resulting in intestinal hypoperfusion and rapid reperfusion leading to epithelial cell damage and loss of epithelial integrity (212). Increased intestinal permeability has been confirmed in athletes (136, 152, 156) and in combat soldiers (110, 125). Whether stress-induced permeability can be prevented or abolished by dietary strategies and whether such intervention would result in relevant end points such as a reduction in stress-associated symptoms remain to be tested.

#### Obesity.

The association between obesity and compromised gut barrier in humans remains an open question. Teixeira et al. observed a slightly, nonsignificantly higher permeability in subjects with obesity (BMI: 35.04 ± 3.98 kg/m^2^, mean ± SE; 201), whereas others found no difference between subjects with overweight/obesity and lean controls (22, 120). Perhaps, intestinal permeability is disturbed only in subjects with morbid obesity. Indeed, subjects with obesity and a BMI ≥40 kg/m^2^ had an elevated intestinal permeability in an LMR test (0.080; 95% confidence interval: 0.073, 0.093) that returned to normal ranges (0.027; 95% confidence interval: 0.024, 0.034) after weight loss despite the subjects still being obese (BMI: 35 kg/m^2^ on average; 38). An alternative explanation for the discrepant results is a lack of sensitivity of the currently used permeability tests. In a recent study, LMRs were not different in a large cohort (*n* = 122) of subjects with severe obesity and nonobese subjects, although ex vivo investigations pointed to subtly compromised barrier function in the subjects with obesity (75).

#### Age.

Impaired barrier function is believed to be a feature of normal aging and to contribute to the increased systemic inflammation observed in elderly people (64). Ex vivo analyses in terminal ileal biopsy tissues from healthy aged (66–77 yr), young (7–12 yr), and adult (20–40 yr) subjects suggested increased small intestinal permeability to solutes in the aged subjects (based on a 2-fold reduction of the transepithelial electric resistance across ileal biopsies in Ussing chambers) with unaffected permeability to macromolecules (134). Nevertheless, urinary excretion of lactulose and mannitol was only slightly lower with increasing age, and the LMR was not different between old and young subjects (141).

#### Smoking.

The effect of cigarette smoking on intestinal barrier function has mainly been investigated in the field of inflammatory bowel diseases (14). Although an initial study suggested that smoking tightens the gut (169), these results were not confirmed in later studies using ^51^Cr-EDTA (12, 169, 195) or PEG (169, 185) as probes.

#### Drugs: NSAIDs and proton pump inhibitors.

NSAIDs are well known to increase intestinal permeability (20). Proton pump inhibitors (PPIs) often are prescribed as a prophylactic treatment against NSAID-induced GI toxicities (177). However, capsule endoscopy revealed exacerbation of NSAID-induced small bowel injury by a PPI (rabeprazole) in 57 healthy subjects (226). In contrast, use of a PPI was not associated with incidence of bloodstream infections in the intensive care unit (data from 24,774 patients), suggesting that PPIs do not meaningfully alter permeability (34).

## HOW CAN WE STRENGTHEN THE PRESENT EVIDENCE THAT DIET IS AN INFLUENCING FACTOR IN THE FUNCTION OF THE INTESTINAL BARRIER IN HUMANS?

The integrated intestinal gut barrier system modulates intestinal exposures so that nutrients, electrolytes, and water are absorbed while simultaneously serving as a first line of defense to protect the host from components found in the environment of the intestinal lumen including both secreted and ingested molecules, as well as gut microbiota and their metabolites. A normal-functioning gut barrier is characterized by a robust physical mucus barrier, intestinal epithelium supporting transcellular and paracellular nutrient transport, and multiple immune factors active in the mucus, in the epithelium, and below the epithelium. Normal function results in nutrient absorption within normal ranges of bioavailability with a balanced immune response such that endogenous and external toxins do not cross the gut barrier, while modulating the immune response to prevent unrestrained inflammation, which results in systemic and bowel inflammatory conditions. Asthma, food allergies, and inflammatory bowel diseases in humans have been associated with the consumption of a Western-style diet high in processed foods and low in fiber (87, 106, 135), suggesting that diet-induced alterations in intestinal barrier function may be playing a role. It remains unknown how much of this dysregulation is due to a definable mechanism that can be linked to barrier function and maintenance of health or subsequent autoimmune and inflammatory chronic diseases in humans.

To identify a pathway forward to strengthen the evidence that diet can be used as a modality to strengthen human intestinal barrier function to reduce the risk of disease, it is fundamentally important to consider the numerous gaps in knowledge that represent hurdles and challenges to this paradigm. On the basis of the evidence described in the previous sections of this article, we provide a summary based on three categories in Table 8.

**Table 8.** Overall concepts and challenges to the paradigm of gut barrier dysfunction

Concept	Challenges to Establishing Role of Gut Barrier Dysfunction
Establishing gut barrier as essential to normal gastrointestinal structure and function in human health	• Dysfunction is best documented in diseases where there is known alteration in histology, anatomy, or function • No evidence yet that repair of intestinal barrier can, by itself, be used to treat a disease in humans • In disease states (e.g., metabolic syndrome), it is inferred that altered barrier function plays a role in disease pathogenesis on the basis of surrogate biomarkers (e.g., LPS or LPS-binding protein in metabolic syndrome), but no direct evidence of causality in humans
Recommending feasible methods to measure normal barrier in human research	• Present quantification of “normal barrier” function in humans is essentially based on measurements in the absence of an association with a disease process • Functionality of measurements is most strongly supported by diseases that alter intestinal mucosal barrier based on histology or anatomy (i.e., IBD, celiac sprue, etc.)
Linking diet to normal gut barrier function among healthy or at-risk people	• The link between diet and normal barrier function has been demonstrated only in precisely controlled and reductionist animal model systems (relevance to human biology unclear) • Small human studies demonstrate improved barrier function in “at-risk” human conditions (e.g., endurance exercise stress, malnutrition, and burns) with dietary supplementation (see Tables 6 and 7)

IBD, inflammatory bowel disease.

Since there are technological limitations in quantifying intestinal barrier function in humans, there is a lack of evidence that reduction of human intestinal barrier function contributes to disease pathogenesis, and there is a lack of evidence that the fortification of barrier function can be used to treat human disease, it is currently tenuous to suggest that diet can be used to fortify human intestinal barrier function with the intent of reducing disease risk.

**Fig. 2.**
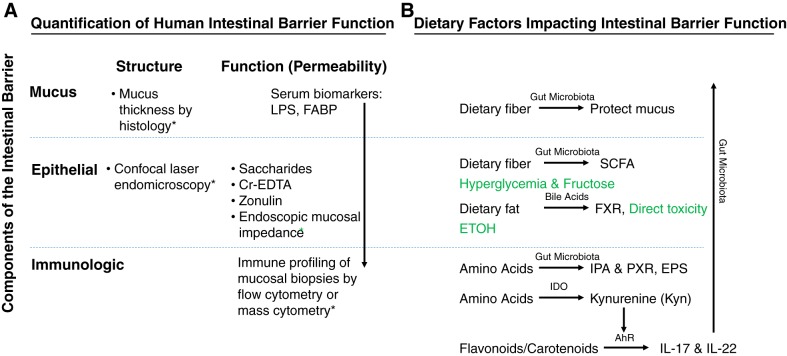
Components of the intestinal barrier. *A*: currently available methods to quantify human intestinal barrier function. *Invasive testing. *B*: dietary factors impacting intestinal barrier function. Green text indicates reduced barrier function with demonstration of relevance in humans. AhR, aryl hydrocarbon receptor; Cr-EDTA, chromium-labeled EDTA; EPS, 4-ethylphenylsulfate; ETOH, ethanol; FABP, fatty acid-binding protein; FXR, farnesoid X receptor; IDO, indoleamine 2,3-dioxygenase 1; IPA, indole-3-propionic acid; PXR, pregnane X receptor; SCFA, short-chain fatty acid.

Given these challenges and gaps in knowledge, what is the approach by which scientific investigation can provide evidence that present notions of how animal model data showing how intestinal barrier function can be reinforced by diet (described in potential of dietary and nondietary factors to impact components of the intestinal mucosal barrier and summarized in Fig. 2) may be relevant to human biology using currently available technologies to quantify human intestinal barrier function (described in measurements to characterize normal structure and function of the intestinal barrier in human nutrition research and summarized in Fig. 2)?

**Fig. 3.**
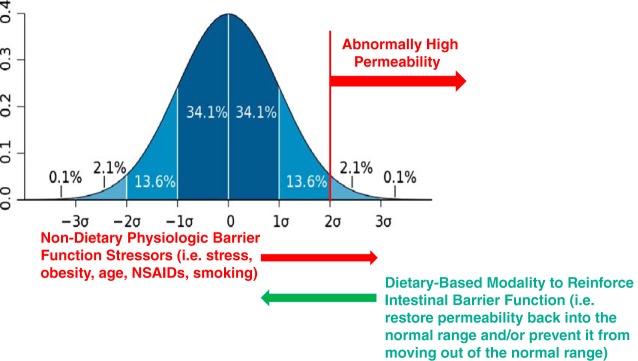
Defining normal boundaries of intestinal permeability as a functional biomarker of barrier function in humans. Studies could be performed in healthy individuals to determine whether the intestinal barrier can be reinforced by diet.

We suggest that the first step is to perform studies to define the boundaries of normal human intestinal barrier function. A reference range defining normal as 95% of values for a test of intestinal permeability using saccharide-based methods (described in measurements to characterize normal structure and function of the intestinal barrier in human nutrition research) in a defined population free from disease would be one approach, if the values fall along a Gaussian distribution. Individuals whose values lay either above or below 2 standard deviations from the mean would be defined as having “abnormal” values (Fig. 3).

However, whether or not this is indicative of a physiologically relevant process (i.e., disease) would need to be established by additional studies designed to determine the sensitivity, specificity, and precision of the test in humans, both with and without disease, known to have an effect on intestinal barrier function as defined by permeability testing. Although individuals with intestinal permeability values that are abnormally high might have an increased risk for the presence or risk of a disease state, the physiological interpretation of an abnormally low permeability measure would be unknown. Assuming that the test was properly performed, perhaps these individuals would have a lower risk for certain disease states, or maybe these individuals should be considered to be normal.

Once the reference range for intestinal permeability has been determined, studies could be designed to assess whether or not specific dietary interventions could prevent, reverse, or improve abnormally high intestinal permeability associated with conditions such as psychological or physical stress, exposure to NSAIDs, age, obesity, and smoking (see potential of dietary and nondietary factors to impact components of the intestinal mucosal barrier and Fig. 3). A positive outcome in such a study might provide evidence that diet can be used to reinforce human intestinal barrier function with the notion that it might reduce the risk for certain diseases. In this fashion, a specific intestinal permeability test could be considered to be a surrogate biomarker of health or disease that is sensitive to diet. Of course, much larger longitudinal prospective cohort studies would need to be performed to validate this notion.

There are many analogies to the approach described above and standard surrogate biomarkers for disease states such as hypertension and blood lipid profiles for the risk of cardiovascular disease and fasting blood glucose and HbA1c levels for type 2 diabetes mellitus. In the latter scenario, abnormally elevated blood glucose and HbA1c levels are associated with either the risk or diagnosis of type 2 diabetes mellitus. Treatment of the prediabetic state or frank diabetes reduces the levels of these two surrogate biomarkers, which is indicative of successful treatment, thereby reducing the risk for morbidity and mortality.

Future research should consider approaches that determine whether dietary interventions help to maintain or restore normal function in healthy populations when exposed to a challenge that affects gut barrier permeability.

## GRANTS

This work was supported by National Institutes of Health Grant R01-DK-115950 to M. Camilleri and by the North American branch of the International Life Sciences Institute (ILSI North America). ILSI North America is a public, nonprofit foundation that provides a forum to advance understanding of scientific issues related to the nutritional quality and safety of the food supply by sponsoring research programs, educational seminars and workshops, and publications. It receives support primarily from its industry membership.

## DISCLAIMERS

The opinions expressed herein are those of the authors and do not necessarily represent the views of the funding organization and the authors’ employers.

## DISCLOSURES

B. J. Lyle provides technical advice and scientific writing consultancy to a range of clients in the private for profit as well as non-profit food and research sector. She is a paid nutrition advisor to the ILSI NA committee that sponsored this project. M. Camilleri: relevant disclosures related to IBS-diarrhea: NIH funding R01-DK115950 and research grants from Novartis (research studies on CLN452) and Allergan (research studies on elobixibat). G. D. Wu: Relevant disclosures related to the gut microbiome and diet include research funding from Seres Therapeutics, Intercept Pharmaceuticals, and Takeda; scientific advisory boards include Danone and Biocodex; consultant agreement with Hitachi. J. Sonnenburg: Cofounder of Novome Biotechnologies, Inc., January, Inc.; scientific advisory board of Clorox/Renew Life, Kaleido Biotechnologies, Second Genome, Gnubiotics Sciences; research funding from Second Genome, Clorox/Renew Life, Abbott Laboratories. Neither of the other authors has any conflicts of interest, financial or otherwise, to report.

## AUTHOR CONTRIBUTIONS

M.C., B.J.L., K.L.M., J.S., K.V., and G.D.W. conceived and designed research; K.L.M. interpreted results of experiments; M.C., B.J.L., K.L.M., J.S., K.V., and G.D.W. prepared figures; M.C., B.J.L., K.L.M., J.S., K.V., and G.D.W. drafted manuscript; M.C., B.J.L., K.L.M., J.S., K.V., and G.D.W. edited and revised manuscript; M.C., B.J.L., K.L.M., J.S., K.V., and G.D.W. approved final version of manuscript.
